# Neurodevelopmental trajectories of cerebellar grey matter associated with verbal abilities in males with autism spectrum disorder

**DOI:** 10.1016/j.dcn.2024.101379

**Published:** 2024-04-09

**Authors:** Jana Klaus, Catherine J. Stoodley, Dennis J.L.G. Schutter

**Affiliations:** a Department of Experimental Psychology, Utrecht University, the Netherlands; b Helmholtz Institute, Utrecht, the Netherlands; c Developing Brain Institute, Children’s National, United States

**Keywords:** ABIDE, Brain development, Crus II, Grey matter volumetry, Language, Verbal ability

## Abstract

Autism spectrum disorder (ASD) is a neurodevelopmental condition frequently associated with structural cerebellar abnormalities. Whether cerebellar grey matter volumes (GMV) are linked to verbal impairments remains controversial. Here, the association between cerebellar GMV and verbal abilities in ASD was examined across the lifespan. Lobular segmentation of the cerebellum was performed on structural MRI scans from the ABIDE I dataset in male individuals with ASD (*N*=144, age: 8.5–64.0 years) and neurotypical controls (*N*=188; age: 8.0–56.2 years). Stepwise linear mixed effects modeling including group (ASD vs. neurotypical controls), lobule-wise GMV, and age was performed to identify cerebellar lobules which best predicted verbal abilities as measured by verbal IQ (VIQ). An age-specific association between VIQ and GMV of bilateral Crus II was found in ASD relative to neurotypical controls. In children with ASD, higher VIQ was associated with larger GMV of left Crus II but smaller GMV of right Crus II. By contrast, in adults with ASD, higher VIQ was associated with smaller GMV of left Crus II and larger GMV of right Crus II. These findings indicate that relative to the contralateral hemisphere, an initial reliance on the language-nonspecific left cerebellar hemisphere is offset by more typical right-lateralization in adulthood.

## 1 Introduction

Autism spectrum disorder (ASD) is a neurodevelopmental condition characterized by impairments in social interaction and communication and the presence of repetitive, stereotyped behaviors (American Psychiatric Association, 2013). In spite of the heterogeneous autism spectrum, the cerebellum is among the brain structures most consistently implicated in ASD (Amaral et al., 2008, Becker and Stoodley, 2013, Bloomer et al., 2022, D’Mello and Stoodley, 2015, Fatemi et al., 2012, Riva and Giorgi, 2000, Sydnor and Aldinger, 2022, Wang et al., 2014). Compared to neurotypical controls, individuals with ASD show atypical cerebellar microstructure and grey matter volumes (GMV) (Duerden et al., 2012, Liloia et al., 2022, Ritvo et al., 1986, Traut et al., 2018) as well as reduced connectivity in cerebello-cerebral networks (Khan et al., 2015, Okada et al., 2022, Olivito et al., 2018, Olivito et al., 2017, Verly et al., 2014). However, recent empirical evidence suggests that the previously proposed association between cerebellar GMV and the phenotype associated with ASD is less straightforward and more complicated than originally thought (Laidi et al., 2022).

A number of studies have attempted to better understand the role of the cerebellum in ASD by investigating associations between cerebellar GMV and specific autism-related symptoms. Reduced GMV of the posterior cerebellum has, for instance, been linked to impairments in social communicative ability in 2- to 13-year old children with ASD (D’Mello et al., 2015, Riva et al., 2013) and social prediction abilities in adults with ASD (mean age: 27 years; Clausi et al., 2021; Olivito et al., 2023). By contrast, two other studies have linked *higher* GMV of posterior lobules VI and VIII and total cerebellar volume to lower social communicative ability (range: 6 – 44 years) (Elandaloussi et al., 2023, Rojas et al., 2006).

Importantly, because ASD is a neurodevelopmental condition, age is likely a relevant factor modulating the relationship between cerebellar GMV and symptomatology. A study in neurotypical children and adolescents showed that in younger children lower GMV in the right posterior cerebellum (Crus II and lobules VIIB, VIIIA and IX) was linked to better reading ability, working memory, and processing speed (Moore et al., 2017), but in older adolescents better cognitive performance was associated with higher GMV of these posterior lobules. Better performance was thus linked to GMV of posterior lobules in an age-dependent manner, suggesting that earlier synaptic pruning in younger children reflects increased brain maturation, which is associated with better cognitive abilities. In the current study, we extrapolated this research question to ASD to examine how cerebellar GMV contributes to verbal abilities as measured by the verbal intelligence quotient (VIQ). Specifically, we addressed the issue of whether developmental trajectories of cerebellar GMV differentially predict verbal abilities in individuals with ASD and neurotypical controls. Similar to previous findings (Moore et al., 2017), we predicted that verbal abilities would demonstrate an age-dependent relationship with posterior cerebellar volumes in neurotypical individuals. The critical question then was whether this relationship would be different in individuals with ASD. In terms of the cerebellar lobules under investigation, we adopted an agnostic approach by including all lobules in the analyses, as functional activation for language processing has been observed in both anterior and posterior lobules of the cerebellum (Guell et al., 2018, Keren-Happuch et al., 2012, King et al., 2019, Stoodley and Schmahmann, 2009, Turker et al., 2023). Thus, including all lobules in the analyses enabled us to investigate potential influences of cerebellar lobules not restricted to the commonly investigated Crus I/II complex. However, based on previous structural findings in ASD cohorts (D’Mello et al., 2015, Riva et al., 2013, Rojas et al., 2006), we expected to see a positive correlation between verbal abilities and GMV in posterior cerebellar structures in children and a negative relationship in adults.

## 2 Methods and materials

### 2.1 Participants

Subjects were selected from the ABIDE I dataset (Di Martino et al., 2014). From the 1112 individuals contained within this dataset, 444 individuals were selected based on the following inclusion and exclusion criteria: (1) availability of a verbal (VIQ) and performance IQ (PIQ) score as measured by a variant of the Wechsler IQ test (WAIS, WASI, WISC); (2) right-handed; (3) male^1^; (4) no current medication intake; (5) no reported psychiatric comorbidity. From this sample, 112 individuals were removed because of insufficient structural scan quality leading to suboptimal segmentation results (see section *Segmentation*). The final sample consisted of 144 individuals diagnosed with ASD (based on either scores from the Autism Diagnostic Observation Scale [ADOS], Autism Diagnostic Interview [ADI] or Social Responsiveness Scale [SRS], clinical expert opinion, or a combination thereof) and 188 neurotypical controls. Table 1 reports demographic and psychometric characteristics by group. Individuals with ASD had significantly lower VIQ scores and marginally lower PIQ scores than the control group.

**Table 1:** Means and SDs (in brackets) for age and IQ measures of the included sample.

	ASD (*N* = 144)			Control (*N* = 188)			
	*M*	*SD*	range	*M*	*SD*	range	Difference
Age	19.3	8.4	8.5 – 64.0	18.4	7.5	8.0 – 56.2	*p* =0.294
VIQ	104.7	16.8	55 – 149	111.1	12.6	80 – 140	*p* <0.0001
PIQ	107.2	14.7	63 – 140	110.1	12.5	67 – 155	*p =*0.066

*Note.* Group differences were assessed with unpaired *t*-tests.

### 2.2 Image acquisition

Structural T1-weighted 3 T MRI scans (1×1×1 mm resolution) were included from twelve scanning sites (i.e., CALTECH, CMU, LEUVEN_1, NYU, PITT, SBL, SDSU, STANFORD, TRINITY, UCLA_1, UCLA_2, USM). Specific scan protocols for each site are available at http://fcon_1000.projects.nitrc.org/indi/abide/abide_I.html.

### 2.3 Cerebellar segmentation

The MRI scans were processed with the CERES pipeline of VolBrain (Romero et al., 2017), an automatic MRI brain volumetry system to parcellate and segment the cerebellum (Carass et al., 2018, Sörös et al., 2021). Within the algorithm, CERES pre-processes T1-weighted MRI scans in Nifti format by denoising, bias field correction, linear registration to the MNI152 template, cropping of the cerebellum, non-linear registration to the cropped MNI152 template, and local intensity normalization. It then labels cerebellar voxels through non-local patch based fusion based on manual segmentations of five high-resolution MR images (Park et al., 2014). GMV measures are then provided relative to total intracranial volume (ICV) for left and right lobules I/II, III, IV, V, VI, VII (Crus I, Crus II, VIIB), VIII (VIIIA, VIIIB), IX, and X, amounting to 24 individual GMV measures.

To ensure adequate quality of the segmentations, all output files were inspected individually. Subjects were excluded from further analyses if the results showed incomplete segmentation of the cerebellum, mislabeling of extracerebellar tissue as cerebellar tissue, visible motion-related artifacts in the MRI scan, or if the MRI scan did not cover the entire cerebellum. This resulted in the exclusion of 38 individuals from the ASD group and 74 from the control group.

### 2.4 Statistical analysis

All statistical analyses were performed in R (version 4.1.2, R Core Team, 2021). To test whether the association between verbal IQ and GMV in cerebellar lobules differs by group membership (ASD vs. neurotypical) and age, we initially fitted a maximal linear mixed effects model with VIQ as the dependent variable. The three-way interactions of group, age, and relative GMV of the 24 cerebellar parcellations (% of total ICV) as well as all lower-order terms were included as fixed effects. A random intercept for scanning site was added to control for potential data quality differences between scanning sites. All continuous variables were *z*-transformed, and the model that best fit the data (quantified by the lowest Akaike Information Criterion value, which estimates the prediction error and selects the model that neither under- nor overfits the data) was determined. In the winning model, normality of residuals and absence of multicollinearity and influential observations were confirmed using the *check_heteroskedasticity, check_multicollinearity,* and *check_outliers* functions in the *performance* package (Lüdecke et al., 2021). Significant interactions were followed up using the *emtrends* function in the *emmeans* package (Lenth, 2021). If these interactions included age, estimated means were calculated at mean ± 1 *SD* (*M* = 18.8 years, *SD* = 7.9, hence 10.9 and 26.7 years, respectively) of the sample age. For convenience, we will hereafter refer to these contrasts as *Children* and *Adults*. Additionally, the winning model was fitted with PIQ as the dependent variable. This allowed us to test the specificity of the obtained model predicting VIQ.

Model outcomes were visualized using a combination of the *sjPlot* (Lüdecke, 2022), *ggplot2* (Wickham, 2016), and *ggpubr* (Kassambara, 2020) packages. The α-level of statistical significance was set to <0.05 (two-tailed). All reported *p* values were FDR corrected for multiple comparisons.

## 3 Results

**Fig. 1:**
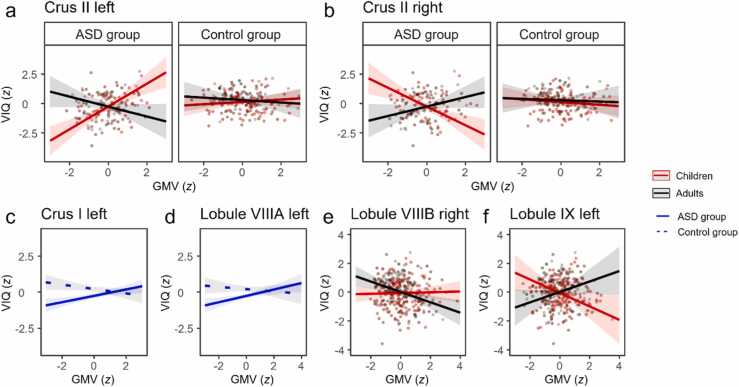
Illustration of significant model terms predicting verbal IQ. Red lines correspond to predicted values for children (i.e., individuals at *M* – 1 *SD* of the sample age, 10.9 years). Black lines correspond to predicted values for adults (i.e., individuals at *M* + 1 *SD* of the sample age, 26.7 years). Solid blue lines refer to the ASD group and dashed blue lines to the control group.

The best model explained 22.2% of the total variance in VIQ scores. Table 2 reports all significant terms of the model with the best fit (see Supplementary Materials, Table S1 for full documentation).^2^ VIQ was predicted by significant three-way interactions of group, age and GMV in left and right Crus II. Higher VIQ was associated with greater GMV of left Crus II in children but smaller GMV in adults with ASD (Fig. 1a). By contrast, for right Crus II, higher VIQ was associated with smaller GMV in children, but larger GMV in adults with ASD (Fig. 1b). No such associations were observed for the control group. Furthermore, higher VIQ was associated with larger GMV of left Crus I and lobule VIIIA in ASD, but smaller GMV of these lobules in the control group (Figs. 1c and 1d). Across groups, higher VIQ was associated with smaller GMV of right lobule VIIIB in adults but not in children (Fig. 1e). Finally, higher VIQ was associated with smaller GMV of left lobule IX in children, but larger GMV in adults (Fig. 1f).

**Table 2:** Model terms from linear mixed model predicting verbal IQ.

Predictors	*β*	*SE*	95% *CI*	*t*	*p*_corr_
Group	-0.47	0.10	-0.68 – -0.26	-4.48	<0.001
VIIIB right	-0.17	0.06	-0.29 – -0.05	-2.70	0.036
Group × Crus I left	0.37	0.11	0.15 – 0.60	3.34	0.013
Group × VIIIA left	0.30	0.12	0.07 – 0.53	2.57	0.036
Age × VIIIB right	-0.19	0.08	-0.35 – -0.04	-2.42	0.048
Age × IX left	0.41	0.16	0.10 – 0.73	2.60	0.036
Group × Age × Crus II left	-0.59	0.22	-1.03 – -0.16	-2.67	0.036
Group × Age × Crus II right	0.57	0.22	0.14 – 1.00	2.62	0.036

Note. *p*_corr_ = FDR corrected *p* values.

To examine whether these findings were specific to verbal abilities, a stepwise backward procedure with PIQ as the dependent variable was performed. In this winning model, no model terms survived multiple comparisons correction (all *p* values > 0.067; see Supplementary Materials, Table S2 for full documentation). Crucially, although there were a number of marginally significant main effects and interactions in this model, we did not observe the same three-way interaction of group, age and GMV of left or right Crus II.

**Fig. 2:**
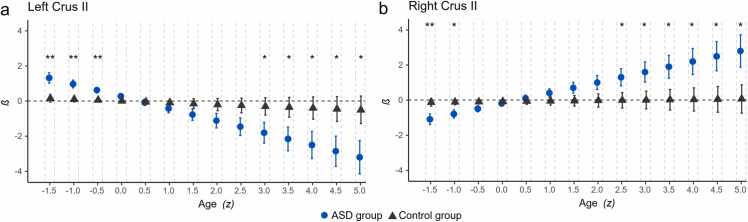
Parameter estimates for the effect of GMV of left (a) and right Crus II (b) across age and between groups (ASD vs. control group) on VIQ. Positive estimates indicate that higher VIQ is associated with higher GMV at a given age; negative estimates indicate that higher VIQ is associated with lower GMV at a given age. Asterisks refer to significant group differences, suggesting a differential developmental trajectory between ASD and control individuals. ** *p* < 0.01, * *p* < 0.05.

To further inspect group-specific trajectories, we extracted the parameter estimates *ß* for the main effects of GMV of left and right Crus II spanning 1.5 *SD* < *M* < 5 *SD* of the sample age to approximate the observed age range of the present dataset (i.e., 6.9–58.4 years) for each group. At each time point, group differences were tested through the interaction of group and GMV of left and right Crus II, respectively (*p* < 0.05, FDR-corrected). The results are shown in Fig. 2 and group differences are documented in Table 3.

**Table 3:** Results for group differences in age-specific GMV effects of left and right Crus II.

Age	*SD*	*β*	*SE*	*t*	*p*_corr_	*β*	*SE*	*t*	*p*_corr_
		Left Crus II				Right Crus II			
6.9	-1.5	1.17	0.34	3.27	0.001	-0.97	0.36	-2.70	0.007
10.9	-1.0	0.87	0.26	3.32	0.001	-0.68	0.27	-2.54	0.012
14.8	-0.5	0.57	0.19	2.96	0.003	-0.40	0.21	-1.93	0.055
18.8	0	0.27	0.18	1.51	0.132	-0.11	0.20	-0.57	0.570
22.7	0.5	-0.02	0.24	-0.10	0.924	0.17	0.25	0.71	0.479
26.7	1.0	-0.32	0.33	-0.98	0.328	0.46	0.33	1.40	0.163
30.7	1.5	-0.62	0.43	-1.44	0.151	0.74	0.43	1.75	0.082
34.6	2.0	-0.92	0.54	-1.70	0.090	1.03	0.53	1.94	0.053
38.6	2.5	-1.22	0.65	-1.87	0.063	1.31	0.64	2.06	0.040
42.5	3.0	-1.51	0.76	-1.98	0.048	1.60	0.75	2.14	0.033
46.5	3.5	-1.80	0.88	-2.06	0.040	1.88	0.86	2.20	0.029
50.5	4.0	-2.10	0.99	-2.13	0.034	2.17	0.97	2.24	0.026
54.4	4.5	-2.40	1.10	-2.17	0.031	2.45	1.08	2.27	0.024
58.4	5.0	-2.70	1.22	-2.21	0.028	2.73	1.19	2.30	0.022

Note. *p*_corr_ = FDR corrected *p* values.

For both left and right Crus II, significant group differences emerged in the youngest individuals with ASD (for left Crus II: between 6.9 and 14.8 years; for right Crus II: between 6.9 and 10.9 years), with left Crus II showing a positive effect on VIQ and right Crus II showing a negative effect. Moreover, with increasing age, group differences were significant from 42.5 to 58.4 years for left Crus II and from 38.6 years to 58.4 years for right Crus II, reflecting the switch in slope reported above.

**Fig. 3:**
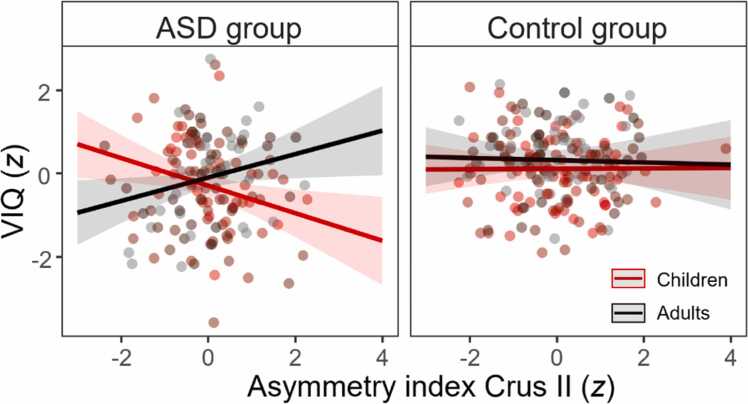
Illustration of the association between Crus II asymmetry and verbal IQ, broken down by group (ASD vs. control) and age. Red lines correspond to predicted values for children (i.e., individuals at *M* – 1 *SD* of the sample age, 10.9 years). Black lines correspond to predicted values for adults (i.e., individuals at *M* + 1 *SD* of the sample age, 26.7 years).

Given the ASD-specific negative relationship between GMV of left Crus II and VIQ and the positive relationship between GMV of right Crus II and VIQ, respectively, we further explored whether this pattern would also translate to group- and age-specific differences when considering an asymmetry index (AI) instead of hemispheric measures. For this, we computed individual AIs for Crus II according to the following formula:$\;\frac{{\mathrm{GMV}}_{\mathrm{right}}-{\mathrm{GMV}}_{\mathrm{left}}}{{\mathrm{GMV}}_{\mathrm{right}}+{\mathrm{GMV}}_{\mathrm{left}}}\;$. Positive values thus reflect a stronger right-ward asymmetry, whereas negative values reflect a stronger left-ward asymmetry. Next, we computed another linear mixed model including the three-way interaction of group, age, and AI as well as all lower-order terms and a random intercept for scanning site and VIQ as the dependent variable. As for the previous models, continuous variables were *z* transformed and the categorical variable of group was sum-coded. In line with the previous findings, VIQ was predicted by a significant three-way interaction of group, age, and AI (*β* = 0.32, *SE* = 0.13, *t* = 2.48, *p*_corr_ = 0.037). In ASD, better verbal abilities were associated with a stronger left-ward asymmetry in children (*β* = -0.33, *SE* = 0.13, *t* = -2.49, *p* = 0.013), while adults showed a positive relationship, indicating that stronger right-ward asymmetry is associated with higher VIQ (*β* = 0.28, *SE* = 0.13, *t* = 2.12, *p* = 0.035; Fig. 3 ). Again, there were no effects in the control group (*p*s > 0.831).

## 4 Discussion

The aim of the present study was to examine the neurodevelopmental trajectory of the relationship between cerebellar GMV and verbal abilities in ASD. An age- and lobule-specific association between cerebellar GMV and verbal IQ in ASD relative to neurotypical controls was observed. Most importantly, verbal abilities were predicted by a distinct age-specific, interhemispheric pattern of GMV in bilateral Crus II in the ASD group: Relative to the control group, better verbal abilities in children were related to a volumetric asymmetry between left and right Crus II (i.e., larger left and smaller right volumes). With increasing age, this association reversed, such that better verbal abilities were associated with relatively larger right, but smaller left Crus II volumes. These findings offer important information regarding age-specific asymmetries between grey matter volumes of cerebellar Crus II and verbal abilities in individuals with ASD.

In this study, all group- and age-specific GMV effects were observed in the posterior cerebellum, echoing findings from neurotypical adults highlighting the relevance of this region for language processing (Guell et al., 2018, Keren-Happuch et al., 2012, King et al., 2019, Stoodley and Schmahmann, 2009). Moreover, individuals with lesions in this region exhibit linguistic impairments like agrammatism, deficits in verbal fluency, and apraxia of speech (Marien et al., 2001, Richter et al., 2007, Silveri, 2021), and better reading and working memory abilities (i.e., skills which are also evaluated by verbal IQ) have been shown to be associated with smaller posterior right cerebellar GMV in neurotypical children, but larger GMV in adolescents (Moore et al., 2017). D’Mello et al. (2016) reported smaller GMV of left Crus I-II in children with ASD and early language delay relative to children with ASD but without early language delay, closely mirroring the patterns we observed in Crus II. Taken together, impaired verbal abilities may be related to atypical Crus II development in childhood (i.e., relatively smaller left Crus II and larger right Crus II volumes). Alternatively, larger volumes of left Crus II in early childhood may allow for compensation of smaller right Crus II volumes by recruitment of right cerebral language regions (D’Mello et al., 2016). Additionally, across groups, age-specific associations were found for right lobule VIIIB and left lobule IX. These regions have been linked to motor/somatosensory functions and working memory and emotion processing (Keren-Happuch et al., 2012, King et al., 2019, Stoodley and Schmahmann, 2009), which arguably are relevant for verbal abilities. Likewise, better verbal abilities were associated with greater GMV of left Crus I and lobule VIIIA in individuals with ASD, regardless of age. The observation that structural properties linked to verbal abilities are not restricted to Crus II can be explained by the fact that verbal IQ is a multifaceted construct representing a number of higher-order functions (e.g., working memory, executive control, processing speed). Moreover, cerebellar functional subregions are not restricted by lobular boundaries, with multiple cortical networks and task activation patterns represented within any given cerebellar lobule (Buckner et al., 2011, King et al., 2019). The present findings thus do not imply that Crus II is exclusively responsible for language processing, but instead indicate that several regions within the (posterior) cerebellum are associated with verbal abilities in ASD in an age-specific manner. Future voxel-level analyses will provide a more detailed evaluation of how the relationship between cerebellar structure and verbal abilities changes developmentally in ASD.

Due to the correlational nature of our study, it is impossible to make any inferences on whether atypical development of cerebellar lobules (i.e., structure) or verbal abilities (i.e., function) are the driving force behind this pattern. That is, atypical cerebellar GMV may be the consequence of a delay in the development of verbal abilities, or the development of verbal abilities may be delayed due to abnormal cerebellar GMV maturation. However, Beuriat et al. (2022) recently argued that in neurotypical individuals, the cerebellum is essential for executive, emotional, and social functions early in life due to insufficiently developed cerebral networks. During brain maturation, the cerebellum then gradually assumes a supportive function. Given the documented cerebello-cerebral hypoconnectivity in ASD (Khan et al., 2015, Okada et al., 2022, Olivito et al., 2018, Olivito et al., 2017, Verly et al., 2014), a more prominent role of dedicated cerebellar structures in verbal abilities might be expected throughout the lifespan in individuals with ASD. This notion is in line with the concept of “cerebellar reserve”, which describes the capacity of the cerebellum to compensate for function loss following, for example, disease-specific neurodegeneration (in other brain regions – diaschisis) (Mitoma et al., 2020), which is assumed to become active around the age of 12 years (Mitoma et al., 2022). Our current understanding of what cerebellar reserve entails is very limited, thus the mechanism proposed here should be considered highly speculative at present. Crucially, the idea of cerebellar compensation does not need to imply that cerebral regions are damaged, less functional, or subject to neuronal degeneration. Rather, the posterior lobules identified in this study may act as a buffering structure to prevent further function loss or to support typical language skills.

A recent study reported that a significant leftward structural lateralisation of the cerebellum in individuals with ASD is associated with stronger deficits in social interaction and communication as measured by the ADI (Li et al., 2023). By extension, this suggests that larger GMV in the right (posterior) cerebellum is linked to better social interaction and communication skills. Following the argument of Beuriat et al. (2022), increased rightward lateralisation in the language-sensitive Crus II as found in the current study may be interpreted as protective against verbal impairment. More specifically, older individuals with higher right-to-left cerebellar GMV outperform those with higher left-to-right GMV. Similar patterns have been found in the cerebral cortex: The right frontal cortex has been shown to be involved in language processing in young neurotypical children (4–6 years) but loses its language-specific functional importance with increasing age (Olulade et al., 2020). Moreover, both children and adults with ASD have been shown to recruit right-hemisphere language homologues in literal language processing, while for neurotypical individuals, this was only seen in children (Williams et al., 2013). In light of this previous research, our findings suggest that the degree of structural cerebellar language lateralisation in ASD is associated with verbal skills in an age-dependent manner. Future research should examine whether these changes in cerebellar asymmetry are associated with more diffuse network formation extending to the cerebral cortex, and whether such changes can be linked to reduced verbal skills.

In the current study, we focused on verbal abilities as assessed by the Wechsler intelligence test. Importantly, next to language impairments, deficits in social communication represent a hallmark symptom of ASD and arguably rely on both linguistic and social abilities. Given the anatomical overlap of activation patterns associated language processing and social cognition primarily in Crus II (Van Overwalle et al., 2014), it is conceivable that similar patterns as those observed here could be extended to social communication. This would help in obtaining further insights into the cerebellar role in ASD symptomatology. Future research could try to link scores from diagnostic measures (e.g., ADOS, ADI) specific to social communication and interaction to volumetric developmental trajectories (D’Mello et al., 2015). Notably, a similar hypothesis has been put forward regarding the role of the posterior cerebellum and social cognition (Van Overwalle et al., 2022). However, this hypothesis focuses on action sequencing and theory of mind underlying social interactions, whereas the current research adds the role of cerebellar Crus II in language processing in ASD.

A number of limitations should be addressed. First, given the cross-sectional nature of the current study, we can only infer differences *between* age groups, while individual developmental trajectories remain elusive. Large-scale longitudinal studies are needed to replicate and extend the present findings. Second, although the age of individuals included in the current study ranged from 8 to 64 years, the sample consisted of relatively few adult individuals. Future research could also focus more on adult individuals with ASD (Lever and Geurts, 2016, Torenvliet et al., 2023) to obtain a complete picture of how cerebellar GMV is linked to ASD symptoms across the lifespan. Third, our analyses are based on chronological age, which is not equivalent to developmental age. Fourth, we only focused on male individuals in this study. Sex differences have been reported in structural cerebellar development for neurotypical individuals (Sussman et al., 2016, Tiemeier et al., 2010) and in GMV in ASD (Chen and Van Horn, 2017, Deng and Wang, 2021), including the cerebellum (Retico et al., 2016, Supekar and Menon, 2015). For these studies, matched male and female ASD and control groups were used, which was not possible in the current study: after applying our exclusion criteria, 49 female individuals qualified, of which 12 individuals with ASD. This underrepresentation of females in large databases of autistic individuals is a common problem, possibly due to diagnostic criteria that favor male diagnoses (D’Mello et al., 2022). As is the case in the majority of studies on ASD, the female sample – compared to 332 male individuals – was thus simply not large enough to systematically investigate potential sex differences. Exploratively, we tested the winning model found for the male sample in the female sample, but none of the model terms were statistically significant (*p*s > 0.865), likely due to low statistical power. Similarly, running the complete model for the female sample resulted in model non-convergence as the number of predictors is larger than the sample size, prohibiting a sound analysis. The findings from the present study therefore cannot be generalized to females with ASD.

In conclusion, verbal abilities in individuals with ASD are associated with age-specific GMV asymmetries in cerebellar Crus II. Findings highlight the necessity to include age as a guiding factor in determining the relevance of the cerebellum in ASD in future studies. Moreover, they provide a structural link to verbal impairments in ASD and delineate the specific contribution of the cerebellum to verbal abilities in ASD across the lifespan.

## CRediT authorship contribution statement

**Catherine Stoodley:** Writing – review & editing, Supervision, Funding acquisition. **Dennis Schutter:** Writing – review & editing, Supervision, Funding acquisition, Conceptualization. **Jana Klaus:** Writing – original draft, Visualization, Project administration, Methodology, Investigation, Formal analysis, Data curation, Conceptualization.

## Declaration of Competing Interest

The authors declare that they have no known competing financial interests or personal relationships that could have appeared to influence the work reported in this paper.

## Footnotes

1 Due to the underrepresentation of female individuals in the ABIDE dataset and the documented differing developmental trajectories of structural cerebellar development (Sussman et al., 2016, Tiemeier et al., 2010), we decided to focus on male individuals only.

2 We here applied a conservative analysis approach by including all cerebellar lobules in the initial model. Given the documented role of Crus I/II in language processing, we also ran a reduced model which only included these lobules as the model terms (i.e., three-way interactions of group, age and GMV for left and right Crus I and Crus II, and all lower-order terms). With all other steps being the same as for the complex analysis, the winning model explained 13.5% of the total variance and included a significant main effect of group (*β* = -0.45, *SE* = 0.11, *t* = -4.18, *p* < 0.001), a significant two-way interaction of group and GMV of left Crus I (*β* = 0.26, *SE* = 0.11, *t* = 2.46, *p* = 0.043), and, crucially, the same three-way interactions of group, age, and GMV of left Crus II (*β* = -0.75, *SE* = 0.22, *t* = -3.37, *p* < 0.001) and right Crus II (*β* = 0.66, *SE* = 0.22, *t* = 3.04, *p* = 0.012). However, model comparison favored the complex model (*Χ*²(13) = 33.08, *p* = 0.002).
