# Supplementary material for: Neurodevelopmental trajectories of cerebellar grey matter associated with verbal abilities in males with autism spectrum disorder
Source: Dev Cogn Neurosci. 2024 Apr 9;67:101379. doi: 10.1016/j.dcn.2024.101379 (PMC11026694; doi:10.1016/j.dcn.2024.101379)
Supplement: Supplementary file 1 — Supplementary material [file mmc1.docx]

**Supplementary material**

Table S1. All model terms of the winning model predicting verbal IQ.

| *Predictors* | *β* | *SE* | *95 % CI* | *t* | *p*_corr_ |
| --- | --- | --- | --- | --- | --- |
| Group | -0.47 | 0.10 | -0.68 – -0.26 | -4.48 | **<0.001** |
| Age | 0.08 | 0.08 | -0.08 – 0.24 | 0.95 | 0.463 |
| Left IV | -0.14 | 0.07 | -0.28 – -0.01 | -2.06 | 0.078 |
| Right IV | 0.14 | 0.07 | 0.00 – 0.27 | 2.03 | 0.078 |
| Right VI | 0.11 | 0.08 | -0.04 – 0.26 | 1.41 | 0.252 |
| Left Crus I | -0.15 | 0.08 | -0.30 – -0.00 | -2.03 | 0.078 |
| Left Crus II | -0.00 | 0.11 | -0.22 – 0.22 | -0.00 | 0.998 |
| Right Crus II | -0.09 | 0.12 | -0.33 – 0.16 | -0.69 | 0.600 |
| Left VIIIA | -0.08 | 0.08 | -0.23 – 0.08 | -1.00 | 0.451 |
| Right VIIIB | -0.17 | 0.06 | -0.29 – -0.05 | -2.70 | **0.036** |
| Left IX | -0.05 | 0.14 | -0.33 – 0.22 | -0.38 | 0.764 |
| Right IX | 0.15 | 0.14 | -0.13 – 0.43 | 1.07 | 0.431 |
| Group × Age | -0.08 | 0.13 | -0.33 – 0.17 | -0.63 | 0.621 |
| Group × Right VI | -0.23 | 0.11 | -0.45 – -0.01 | -2.06 | 0.078 |
| Group × Left Crus I | 0.37 | 0.11 | 0.15 – 0.60 | 3.34 | **0.013** |
| Group × Left Crus II | 0.27 | 0.17 | -0.07 – 0.62 | 1.57 | 0.197 |
| Age × Left Crus II | -0.10 | 0.14 | -0.38 – 0.18 | -0.71 | 0.600 |
| Group × Right Crus II | -0.11 | 0.19 | -0.48 – 0.26 | -0.60 | 0.621 |
| Age × Right Crus II | 0.03 | 0.14 | -0.25 – 0.31 | 0.20 | 0.872 |
| Group × Left VIIIA | 0.30 | 0.12 | 0.07 – 0.53 | 2.57 | **0.036** |
| Age × Left VIIIA | -0.16 | 0.07 | -0.30 – -0.01 | -2.15 | 0.078 |
| Age × Right VIIIB | -0.19 | 0.08 | -0.35 – -0.04 | -2.42 | **0.048** |
| Age × Left IX | 0.41 | 0.16 | 0.10 – 0.73 | 2.60 | **0.036** |
| Age × Right IX | -0.34 | 0.16 | -0.65 – -0.03 | -2.13 | 0.078 |
| Group × Age × Left Crus II | -0.59 | 0.22 | -1.03 – -0.16 | -2.67 | **0.036** |
| Group × Age × Right Crus II | 0.57 | 0.22 | 0.14 – 1.00 | 2.62 | **0.036** |
| *Random Effects* | | | | | |
| *σ*^2^ | 0.77 | | | | |
| *τ*_00site_ | 0.01 | | | | |
| ICC | 0.02 | | | | |
| *N*_site_ | 12 | | | | |
| Observations | 332 | | | | |
| Marginal *R*^2^ / Conditional *R*^2^ | 0.208 / 0.222 | | | | |

Table S2. All model terms of the winning model predicting performance IQ.

| *Predictors* | *β* | *SE* | *95 % CI* | *t* | *p*_corr_ |
| --- | --- | --- | --- | --- | --- |
| Group | -0.26 | 0.11 | -0.48 – -0.04 | -2.32 | 0.078 |
| Age | 0.18 | 0.09 | 0.01 – 0.35 | 2.05 | 0.103 |
| Left I-II | -0.20 | 0.10 | -0.41 – 0.00 | -1.97 | 0.110 |
| Right I-II | 0.07 | 0.10 | -0.13 – 0.27 | 0.70 | 0.653 |
| Right IV | 0.01 | 0.08 | -0.15 – 0.17 | 0.10 | 0.922 |
| Right V | 0.07 | 0.09 | -0.11 – 0.24 | 0.75 | 0.653 |
| Left VI | -0.06 | 0.07 | -0.19 – 0.07 | -0.94 | 0.549 |
| Left Crus II | 0.05 | 0.08 | -0.11 – 0.21 | 0.65 | 0.653 |
| Left VIIB | 0.05 | 0.07 | -0.09 – 0.18 | 0.67 | 0.653 |
| Right VIIIB | 0.03 | 0.07 | -0.11 – 0.17 | 0.38 | 0.804 |
| Right IX | -0.02 | 0.06 | -0.15 – 0.10 | -0.39 | 0.804 |
| Left X | -0.10 | 0.08 | -0.25 – 0.06 | -1.22 | 0.412 |
| Group × Age | -0.15 | 0.14 | -0.44 – 0.13 | -1.07 | 0.476 |
| Group × Left I-II | 0.08 | 0.19 | -0.30 – 0.46 | 0.39 | 0.804 |
| Group × Right I-II | 0.02 | 0.18 | -0.34 – 0.38 | 0.11 | 0.922 |
| Group × Right IV | 0.15 | 0.13 | -0.09 – 0.40 | 1.23 | 0.412 |
| Group × Right V | -0.26 | 0.12 | -0.49 – -0.02 | -2.11 | 0.103 |
| Group × Left Crus II | 0.08 | 0.12 | -0.15 – 0.31 | 0.66 | 0.653 |
| Group × Right VIIIB | -0.27 | 0.11 | -0.49 – -0.04 | -2.32 | 0.078 |
| Group × Left X | 0.28 | 0.12 | 0.06 – 0.51 | 2.46 | 0.078 |
| Age × Left I-II | 0.02 | 0.10 | -0.18 – 0.22 | 0.20 | 0.898 |
| Age × Right I-II | -0.18 | 0.12 | -0.41 – 0.06 | -1.47 | 0.296 |
| Age × Right IV | -0.02 | 0.08 | -0.18 – 0.13 | -0.28 | 0.856 |
| Age × Right V | -0.21 | 0.07 | -0.35 – -0.07 | -2.93 | 0.067 |
| Age × Left VI | 0.23 | 0.09 | 0.05 – 0.40 | 2.55 | 0.074 |
| Age × Left Crus II | -0.17 | 0.08 | -0.33 – -0.02 | -2.17 | 0.101 |
| Age × Left VIIB | 0.21 | 0.08 | 0.06 – 0.36 | 2.70 | 0.067 |
| Age × Right IX | -0.16 | 0.06 | -0.28 – -0.04 | -2.67 | 0.067 |
| Group × Age × Left I-II | -0.52 | 0.22 | -0.96 – -0.09 | -2.40 | 0.078 |
| Group × Age × Right I-II | 0.57 | 0.20 | 0.17 – 0.96 | 2.82 | 0.067 |
| Group × Age × Right IV | 0.26 | 0.13 | 0.01 – 0.50 | 2.03 | 0.103 |
| Group × Age × Left Crus II | -0.23 | 0.11 | -0.46 – -0.01 | -2.06 | 0.103 |
| *Random Effects* | | | | | |
| *σ*^2^ | 0.82 | | | | |
| *τ*_00site_ | 0.00 | | | | |
| ICC | 0.00 | | | | |
| *N*_site_ | 12 | | | | |
| Observations | 332 | | | | |
| Marginal *R*^2^ / Conditional *R*^2^ | 0.178 / 0.182 | | | | |
